# Anti-COVID-19 Vaccination Alters the Menstrual Cycle and Dose Accumulation Enhances the Effect

**DOI:** 10.3390/medicina60060956

**Published:** 2024-06-08

**Authors:** Hospital Español de Pachuca Research Group, Roque D. Licona-Meníndez, Alberto N. Peón

**Affiliations:** 1 Hospital Español de Pachuca, Pachuca 42088, Mexico; 2 Sociedad Española de Beneficencia, Pachuca 42060, Mexico

**Keywords:** SARS-CoV-2, COVID-19, vaccines, vaccination, menstrual cycle alterations, menstrual cycle

## Abstract

*Background and Objectives*: New investigations have detected an enhanced probability for women to develop menstrual cycle alterations after anti-COVID-19 vaccination. Moreover, given that the protective immunity provided by anti-COVID-19 vaccination appears to wane quickly, booster vaccination has been recommended. Nonetheless, whether adverse events arise from such repeated immunization has not been studied. *Materials and Methods*: We studied the incidence of menstrual cycle alterations, the quantity of menstrual cycle alterations per subject, and of altered menstrual cycles in nonpregnant women of fertile age after anti-COVID-19 vaccination in a cohort of vaccinated female subjects by the means of a standardized questionary that was applied via telephone calls each month. Subjects that received up to four doses were studied for 6 months after each dose. We calculated the odds ratio for enhanced incidence, as well as quadratic functions for the tendencies. A sensitivity analysis excluding subjects taking hormonal birth control and those with polycystic ovary syndrome was performed. *Results*: Anti-COVID-19 vaccination enhanced the probability to develop menstrual cycle alterations (OR 1.52, CI at 95% 1.2–1.8, *p* < 0.0001) and, interestingly, such a tendency was enhanced when subjects received more doses (R^2^ = 0.91). Furthermore, the same trends repeated for the quantity of alterations per subject, and of altered cycles. Such an effect was further demonstrated to be independent upon the vaccine brand being applied, the birth control status, and the diagnosis of polycystic ovary syndrome. *Conclusions*: Vaccination is the most cost-effective measure for primary prevention and is considered to be safe. Nonetheless, in this article, we show data that suggest that repeated vaccination of adult female subjects may lead to an enhanced incidence of menstrual cycle–related adverse events, quantity of alterations per subject, and altered cycles. We therefore think that the development of new vaccine formulations that produce longer-lasting immunity is of paramount importance to reduce the potential for dose accumulation–dependent enhanced risk.

## 1. Introduction

Vaccines have been essential tools for public health since their inception in 1796 [1], and as vaccination campaigns are developed and new vaccine formulations are made, more people benefit from their properties each year. For example, the vaccination efforts that were performed between 2000 and 2020, against only 10 diseases, were estimated to have saved 50 million lives [2]. And, interestingly, the vaccination efforts made a year later, against only COVID-19, were estimated to have prevented 14.4 million additional deaths [3]. In such a scenario, the formulation of new vaccines and the design of better vaccination campaigns is of paramount importance for public health.

However, protection against both laboratory-confirmed infection and symptomatic COVID-19 have been described to significantly wane 6 months after the primary vaccination cycle [4], and although the protection against hospitalization and death has remained significantly high after a year [5], concern has been raised in regard to waning immunity. Thus, booster doses have been recommended after 4 to 6 months by the Centers of Disease Control [6], and such recommendation has been in operation in several countries.

In addition to the natural waning of immunity, the emergence of new variants has also challenged vaccine efficacy [7], raising a concern about long-term protection from available vaccines, which are all designed to provide protection against the original Wuhan variant. In such circumstances, the idea of developing strain-specific or multistrain vaccines has also been proposed [8]. However, in the current circumstances, booster immunization is a necessity.

On the other hand, anti-COVID-19 vaccines are generally considered to be safe, but nonsevere adverse events have been reported [9] nonetheless. Among these, menstrual cycle alterations (MCAs) stand out, which are a matter of intense debate [10,11], but the hypothesized mechanisms have a good rationale and there is accumulating evidence for this phenomenon [11,12]. For instance, it has been proposed that vaccines have an impact on the hypophysis–hypothalamus axis, leading to an enhancement of cortisol secretion, and thus to a systemic immune dysregulation that impacts the uterine milieu. Moreover, it is thought that the immunologic impact of vaccines may cause a local dysbiosis, which may alter the inflammatory and endocrine characteristics of the uterus [13].

As the alteration of the menstrual cycle has been previously studied, in the present work, we not only asked whether anti-COVID-19 vaccines would be able to induce MCAs in nonpregnant women at a fertile age, but if a booster-based vaccination campaign would be more prone to enhance the probability for developing MCAs than the normal two doses that have been recommended and vastly studied. We found that the probability to present MCAs is significant from the first dose, but it is enhanced by further doses. This same phenomenon occurs with the quantity of MCAs per subject, and the endurance of the alterations after receiving each dose. This effect does not appear to be dependent on the vaccine type or brand, and a sensitivity analysis failed to show that the alterations occurred in dependence of polycystic ovary syndrome or hormonal birth control use.

## 2. Materials and Methods

We interviewed 522 female subjects at a fertile age who were not pregnant or lactating, had both ovaries and uterus, and were vaccinated against COVID-19 with any vaccine brand between May 2021 and June 2023. All participants were residents of the state of Hidalgo, México, and signed an informed consent form and agreed to a telephonic follow-up date monthly for 6 months after each dose. Each subject was evaluated for (i) incidence of alterations, (ii) number of alterations per subject, (iii) duration of alterations, and (iv) type of alterations. All the subjects were recruited after the application of their first dose at the Hospital de la Sociedad Española de Beneficencia, Pachuca, Hidalgo, México. We utilized a standardized questionary for all the interviews, which was written in Spanish, and was not validated. Nonetheless, expert physicians answered the questionary after the examination of the subjects.

MCAs were defined as self-reported disturbances of period timing (before or after the expected moment), and duration of the cycle and of the bleeding; the occurrence of unexpected pain, intermenstrual bleeding, changes in period flow, and amenorrhea. The control group consisted of the menstrual condition of the subjects before being vaccinated with the first dose.

We studied female subjects that met the following inclusion criteria: fertile age (18–45 years old) who were not pregnant or lactating, had both ovaries and uterus, and were vaccinated against COVID-19 with any vaccine brand. Also, participants with any of the following exclusion criteria were discontinued from this study: cancer, malnutrition, and not meeting inclusion criteria.

The odds ratios (ORs) with a confidence interval (CI) at 95% of increased probability to present an MCA were calculated for each dose and each vaccine brand administered in every dose, and such data were plotted into a Forest plot to calculate the overall odds for developing an MCA. The tendency for an enhancement in the overall incidence with accumulating vaccine doses was evaluated with a quadratic regression using the average incidence for each dose. Moreover, the quantity of alterations per subject and of altered cycles after each dose were averaged, and a standard deviation was also calculated. Both were plotted into histograms and significant differences were detected using a paired Mann–Whitney U test. These data were also analyzed in a whisker plot with a mixed-effects model. Finally, the average incidence, quantity of alterations per subject, and duration of alterations were plotted into a multivariate analysis.

All the figures and the statistic calculations were performed with GraphPad Prism 9, and the threshold for significant differences was *p* ≤ 0.05.

Because polycystic ovary syndrome and hormonal birth control were thought to act as potential confounders, we performed a sensitivity analysis subtracting the data from subjects with either or both conditions.

We obtained the ethical approval for this study on June 12th 2020 from the “Comité de Ética en Investigación de la Sociedad Española de Beneficencia,” and all the subjects that formed part of this study signed an informed consent form. All the procedures were carried according to the Declaration of Helsinki.

## 3. Results

We studied 522 female subjects at a fertile age who were not pregnant or lactating, had both ovaries and uterus, and were vaccinated against COVID-19 with any vaccine brand between May 2021 and June 2023. All the subjects contributed to the control group with their prevaccination status data, as well as with data for the first dose. Of the subjects, 497 (95.2%) received a second dose and contributed with their data, while 395 (75.6%) received a third and 128 (24.5%) received a fourth, adding up to 1542 doses overall (Supplementary Figure S1). From the total of 1542 doses, 638 (41.3%) were AstraZeneca, 456 (29.5%) were Pfizer, 194 (12.5%) were Sinovac, 113 (7.3%) were Cansino, 73 (4.7%) were Moderna, and 68 (4.4%) were the Sputnik brand (Supplementary Table S1).

**Figure 1:**
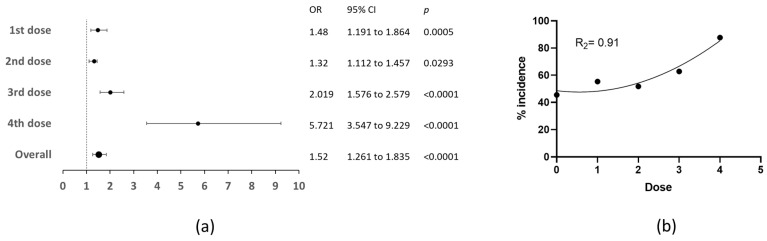
The incidence of menstrual cycle alterations increases by dose accumulation. (**a**) Both the odds ratios for increased incidence of menstrual alterations in each dose, (**b**) and tendency of such incidence to be enhanced with dose accumulation were investigated. OR, odds ratio; CI, confidence intervals. The R^2^ was calculated over a quadratic function.

Overall, the incidence of MCAs is significantly increased by anti-COVID-19 vaccination (OR 1.52, CI at 95% 1.2–1.8). Interestingly, while it is enhanced from the first (OR 1.48, CI at 95% 1.191 to 1.864, *p* = 0.0005) and second (OR 1.32, CI at 95% 1.112 to 1.457, *p* = 0.0293) doses, the effect size is more conspicuous at the third (OR 2.019, CI at 95% 1.576 to 2.579, *p* < 0.0001) and, especially, the fourth (OR 5.7, CI at 95% 3.547 to 9.229, *p* < 0.0001) doses (Figure 1a).

Upon such observation, we calculated a quadratic regression over the main incidence of MCAs for each dose, finding an enhanced probability (R^2^ = 0.91) for developing MCAs with dose accumulation, in an effect that became evident at dose three to four (Figure 1b). As such, the dose accumulation–dependent enhanced risk (DADER) effect over the incidence of MCAs became apparent.

Because the population that we studied had a heterogeneous composition in terms of vaccine brands applied, we investigated whether any of the particular formulations were responsible for the effect that we detected for dose accumulation. To research this phenomenon, we calculated the OR and 95% CI of each vaccine brand in every dose applied, and determined that on the first dose, the Sinovac vaccine was the only drug with the potential to induce an increased probability to develop an MCA (OR 1.84, CI at 95% 1.107–3.065), while the Pfizer vaccine was the only one that apparently worked as a protective factor (OR 0.57, CI at 95% 0.36–0.89) (Supplementary Figure S2a). Moreover, for the vaccines applied at the second dose, both Moderna (OR 2.2, CI at 95% 1.1–4.4) and Pfizer (OR 1.48, CI at 95% 1.03–2.31) increased the probability of suffering an MCA (Supplementary Figure S2b).

Sinovac (CI 3.8, CI at 95% of 1.5 to 10), Cansino (OR 3.2, CI at 95% 1.6–6.2), and AstraZeneca (OR 3, CI at 95% 2–4.7) vaccines correlated with enhanced probabilities for developing an MCA at the third dose (Supplementary Figure S2c). And finally, Moderna (CI 3.1 CI at 95% of 1.08–9.4), Sinovac (OR 6.7, CI at 95% 2.1–21.4), Sputnik (OR 5.2, CI at 95% 1.29–21.3), Cansino (OR 5.3, CI at 95% 1.8–14.8), Pfizer (OR 5.1, CI at 95% 2.2–12), and AstraZeneca (OR 8.3, CI at 95% 2.9–22.4) vaccines correlated with an enhanced probability for developing an MCA at the fourth dose (Supplementary Figure S2d).

To further discard a specific vaccine brand–dependent artifact, we performed a linear regression with a 95% CI over the mean incidence of MCAs for each brand, starting at the control group and progressing with data from the other doses. Both Sinovac and AstraZeneca vaccines had an R^2^ = 0.91, followed by Cansino with R^2^ = 0.89, Sputnik with R^2^ = 0.84, and Moderna with R^2^ = 0.73 (Supplementary Figure S2). Only the Pfizer vaccine had a nonsignificant R^2^ = 0.40, but followed the same pattern as the others. This analysis confirmed the tendency for each vaccine brand to induce a DADER.

To address for potential confounders, we calculated the OR for enhanced MCAs at each dose excluding the subjects with polycystic ovary syndrome (POS) (Supplementary Table S2), as well as those using hormonal birth control (HBC) (Supplementary Table S3). An enhanced probability for an elevated MCA incidence was observed again at the third and fourth doses in both cases, confirming that the accumulation of doses, disregarding the vaccine brand or the POS or HBC status of the subject, induces an enhancement of an MCA incidence.

**Figure 2:**
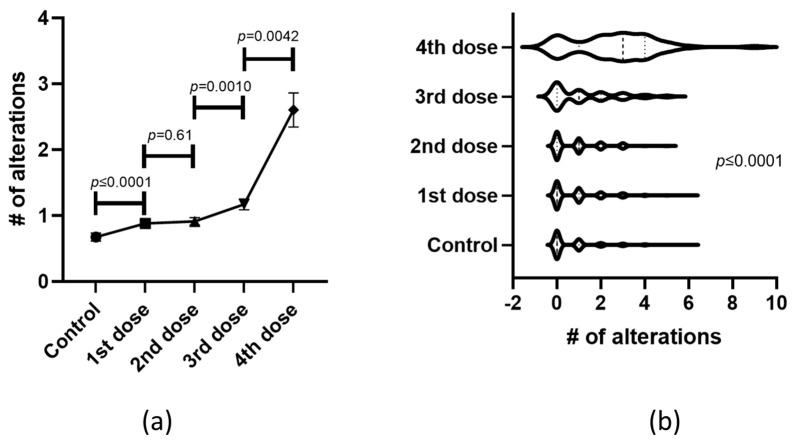
The number of alterations increases as more doses accumulate. (**a**) The number of alterations per subject was averaged and plotted into a histogram and significant differences were detected using a Mann-Whitney U test. (**b**) The differences were plotted into a violin plot and significant differences were calculated with a mixed-effects model.

Moreover, the mean and SD for the number of alterations induced on each dose were calculated and plotted into a histogram, showing that dose accumulation increases the risk for developing an enhanced quantity of alterations per subject (Figure 2a). A further analysis of such data with a violin plot and a mixed-effects model revealed that the first two doses did not produce a significant impact on the quantity of MCAs, but the violins became significantly (*p* ≤ 0.0001) widened toward the third and, especially, the fourth doses (Figure 2b), suggesting that more subjects presented more MCAs as an effect of anti- COVID-19 dose accumulation.

**Figure 3:**
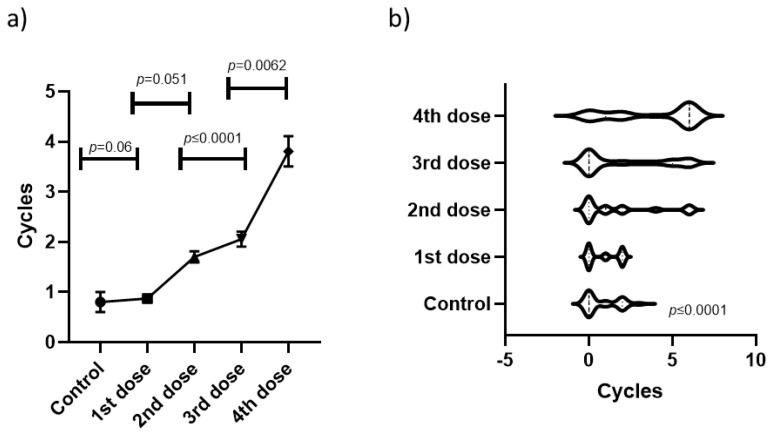
The duration of alterations increases as more doses accumulate. (**a**) The duration of alterations per subject was averaged and plotted into a histogram and significant differences were detected using a Mann-Whitney U test. (**b**) The data were plotted into a violin plot and significant differences were calculated with a mixed-effects model.

**Figure 4:**
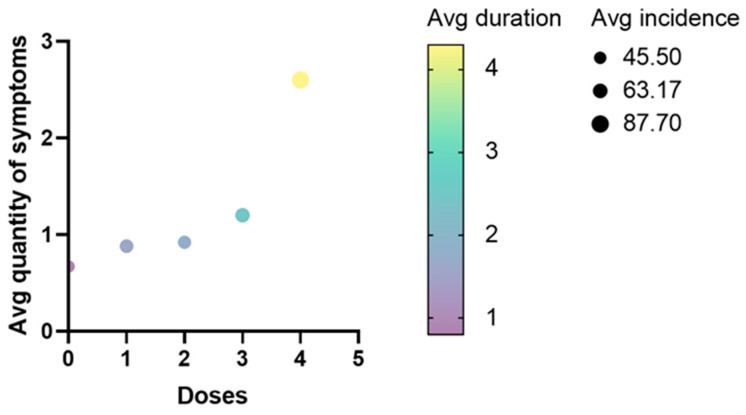
Multivariate analysis. The average incidence, quantity of altered cycles, and quantity of symptoms per dose were plotted into a multivariate analysis.

Additionally, we measured the mean quantity of altered cycles, finding that the third (*p* ≤ 0.0001) and fourth (*p* ≤ 0.0062) doses, but not the first and second, were able to significantly increase the quantity of months that the subjects remained with an altered menstrual cycle (Figure 3a). A further analysis through violin plots confirmed that the MCA that lasted 4 to 6 months only became more common at the third and fourth doses (Figure 3b). Furthermore, a multivariate analysis qualitatively showed that the accumulation of anti-COVID-19 vaccine doses causes enhancements on the three evaluated parameters (average incidence, quantity of altered months, and quantity of symptoms) at the same time (Figure 4).

## 4. Discussion

Our data revealed that anti-COVID-19 vaccination in nonpregnant women of a fertile age with different vaccine brands such as AstraZeneca, Pfizer, Cansino, Sputnik, Sinovac, and Moderna is able to enhance the incidence of MCAs, which includes altered timing and volume of menstruation, as well as pain, duration of the bleeding, period loss, and intermenstrual bleeding. Such effect can be observed from the first to the fourth doses, and in a global ponderation. And, importantly, the effect became more pronounced when more doses were applied. There are numerous confounding factors to our study, such as body–mass ratio, coexistence with other health conditions, time of vaccination (how far from the menstrual cycle when injected), the subjects’ ability to recollect events related to the MCA, and the subjectivity of the response. And this fact can affect the evaluation of parameters tested in this study. Therefore, a more comprehensive study on MCAs using a large sample size is warranted to causally establish the link (if any) between anti-COVID-19 vaccination and MCAs. Nonetheless, taking these considerations into account, a sensitivity analysis was made excluding the subjects with polycystic ovary syndrome or hormonal birth control, and the results were still significant, so we can only conjecture about the potential participation of the other confounding factors.

To our notice, only one study has found that COVID-19 vaccination does not relate to an increased incidence of MCAs [14]; nonetheless, this study analyzed only one variable related to MCAs, which is the timing. In other studies, with more comprehensive designs, an increase in an MCA incidence in relation to vaccines has been detected to be as high as 53.7% [15] and, importantly, a recent systematic review and meta-analysis showed a significant association between MCAs and anti-COVID-19 vaccination in 25,054 women of reproductive age (OR 1.91, CI at 95% 1.76–2.07) [16]. These findings are consistent with the first discovery of our study, which is the increased incidence of MCAs (OR 1.52, CI at 95% 1.26–1.83).

Other studies have found that while anti-COVID-19 vaccination is able to induce an enhanced incidence of MCAs, the vaccine brand did not influence such outcome [17]. This is paralleled by our research, which shows that every vaccine brand has increased potential to induce an MCA.

Interestingly, research by Laganà and colleagues found an MCA incidence of 50–60% for the first anti-COVID-19 vaccine and an enhanced incidence of 60–70% for the second dose [18], while another study made by Alvergne et al. found that the vaccine-enhanced MCA dissipates in unvaccinated cycles [19]. To better understand this phenomenon, we studied the DADER over four vaccine doses and their respective follow-up period, accounting for a total of 30 months (6 months after each dose), showing a significant tendency for the increase in MCA incidence when more doses were applied. In addition to incidence, we measured the quantity of symptoms per subject and the duration of the symptomatology after an applied dose. We found that in parallel to the incidence, the quantity of symptoms per subject and the duration of the symptoms postvaccination increased when more doses per subject were accumulated, not always reaching a dissipation in unvaccinated cycles as seen in Alvergne et al. [19].

Anti-COVID-19 vaccines were raised in an emergency-use authorization context, and information about their long-term security and efficacy is lacking. Nonetheless, a systematic review and metaregression study published by *The Lancet* shows that during 6 months after full vaccination, vaccine efficacy against symptomatic COVID-19 decreased by approximately 20–30% for most vaccines. However, it has been shown that vaccine efficacy decreases more against infection and symptomatic disease than against severe disease in the first 6 months after full vaccination. In fact, most studies showed that vaccine efficacy against severe disease was maintained above 70% after full vaccination (at least two doses), with a minimal decrease after 6 months. Thus, vaccination plans including up to two doses and two boosters have been promoted. Despite this, the data hereby gathered raise a question about the safety of repeated immune-stimulation [20].

On the other hand, recent research has shown that the menstrual cycle is tightly regulated by neuro–immune–endocrine pathways in such a way that it is unsurprising to observe the dysregulation of such a process by immunological phenomena, like vaccination. For instance, a Th2-like immune profile has been associated with estrogen production, while progesterone has been detected to corelate with a Th1-type immune profile [21]. And, interestingly, these changes are mirrored by an enhanced expression of tumor necrosis factor-α (TNF-α), interleukin-1β (IL-1β), IL-6, and IL-8 in the endometrium of female subjects undergoing the late proliferative phase, when progesterone levels are dominant [22]. In fact, Guterstam and colleagues [23] found that the endometrial cytokine microenvironment is distinct from that of the blood, and that in frank contrast with the plasma levels of cytokines, which remain constant, IL-1β and IL-6 levels increase in the late luteal phase in association with progesterone.

Moreover, the effect of estrogen over, at least, TNF-α production is dose dependent, because low concentrations of it (like those seen throughout the menstrual cycle) stimulate this cytokine production, but enhanced levels (like those seen in pregnancy) are able to suppress its production [24]. This observation has been mirrored by experimental treatments with estrogen to lower TNF-α in the setting of multiple sclerosis [25]. Nonetheless, the TNF-α-estrogen axis may be bidirectional, because the treatment with the first is able to induce an enhancement in the levels of the latter, as well as the conversion rate of E1 (estrone) into E2 [26].

On the other hand, the expression of both hormone and neuropeptide receptors have been detected on immune cells and the expression of cytokine receptors have been reported on both gland and neural cells, in such a way that the change in the secretion pattern of one system is thought to impact in the other components of the neuro–immune–endocrine system [27]. In fact, alterations in the whole neuro–endocrine networks have been described in relation to immunological phenomena, like vaccination [28]. As such, other authors have thought that the changes related to anti-COVID-19 vaccination in the regulation of the menstrual cycle may be dependent upon the immune response to vaccination, rather than a specific vaccine component having a direct effect on the endocrine mechanisms [29].

Moreover, another hypothesis relates the impact of vaccines in the hypothalamus–hypophysis axis with an enhanced systemic cortisol production, which can be associated with changes in many physiological processes, like the menstrual cycle [13]. And finally, other authors have proposed an important role for the chronic psychological stress of the lockdown, with the induction of hypothalamic hypogonadism through the kisspeptinergic system [30], but we think that, in this instance, it is clear that the administration of an immunological stressor is key for the induction of this phenomenon, so that we do not think that the psychological stress may have a significant effect on the regulation of the menstrual cycle after vaccination.

Taken together, these investigations lead to the hypothesis that the immunological effects of anti-COVID-19 vaccination may induce changes in the immunological aspect of menstrual cycle regulation, potentially explaining the phenomenon seen in this report. Nonetheless, we lack data to confirm such hypothesis and more research is needed to provide a refined understanding of this phenomenon, because we also lack understanding about the potential implications that an infection with COVID-19 may have on the whole phenomenon. Such understanding is important, for instance, because whether anti-COVID-19 vaccination effects on the regulation of the menstrual cycle induce fertility decay or other long-term problems is unknown, but the hypothesis has good rationale [31,32,33].

## 5. Conclusions

In the present study, we appreciate the presence of postvaccination menstrual disturbances in women, and while it is undebatable that vaccination prevents severe illness and death, being the most efficacious sanitary measure that contributes to public health, it may also be true that newer vaccine formulas or enhanced vaccination plans may be needed in order to avoid the effects of the DADER that repeated vaccination represents. Thus, the development of new vaccine formulas that generate enduring immunological memory should be considered as important research topics for the near future.

## Data Availability

Data is unavailable due to privacy protection policies.

## Supplementary Materials

The following supporting information can be downloaded at: https://www.mdpi.com/article/10.3390/medicina60060956/s1, Table S1. Treatment descriptors. Table S2. Sensitivity analysis excluding polycystic ovary syndrome. Table S3. Sensitivity analysis excluding subjects using hormonal birth control. Figure S1. Participants flowchart. Figure S2. The enhanced probability for menstrual cycle anomalies with dose accumulation is independent from the type of vaccine received. The odds ratios and confidence intervals for increased incidence of menstrual cycle alterations in relation to individual vaccine brands were measured after the first (a), second (b), third (c) and fourth (d) doses. Figure S3. Individual tendency for vaccine-brands in the increased incidence of menstrual cycle alterations with dose accumulation. A linear regression with a 95% confidence interval to describe the tendency to induce enhanced incidence of the MCA with dose accumulation was calculated for each vaccine brand, including Sinovac (a), AstraZeneca (b), Cansino (c), Pfizer (d), Moderna (e), and Sputnik (f). Full list of author names (collaborators on Hospital Español de Pachuca Research Group).
