# Supplementary material for: Anti-COVID-19 Vaccination Alters the Menstrual Cycle and Dose Accumulation Enhances the Effect
Source: Medicina (Kaunas). 2024 Jun 8;60(6):956. doi: 10.3390/medicina60060956 (PMC11206152; doi:10.3390/medicina60060956)
Supplement: Supplementary file 1 [file medicina-60-00956-s001.zip › medicina-2973181-supplementary.pdf]

Supplementary Table S1. Treatment descriptors.

						% of total	% of each dose per brand			
	1st Dose	2nd Dose	3rd Dose	4th Dose	Total doses	% per brand	1st Dose	2nd Dose	3rd Dose	4th Dose
**AstraZeneca**	170	191	211	66	638	41.3	32.5	38.4	53.4	51.5
**Pfizer**	191	174	67	24	456	29.5	36.5	35.0	16.9	18.7
**Sinovac**	71	64	49	10	194	12.5	13.6	12.8	12.4	7.8
**Moderna**	20	33	12	8	73	4.7	3.8	6.6	3.0	6.2
**Sputnik**	24	20	16	8	68	4.4	4.5	4.0	4.0	6.2
**Cansino**	46	15	40	12	113	7.3	8.8	3.0	10.1	9.3
**Total**	522	497	395	128	1542					
%	100	95.2	75.6	24.5		100	100	100	100	100

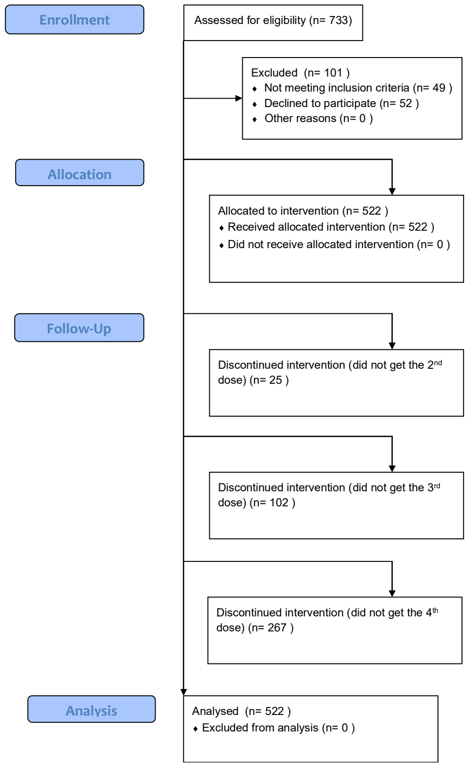

Supplementary Figure S1. Participants flowchart.

a)

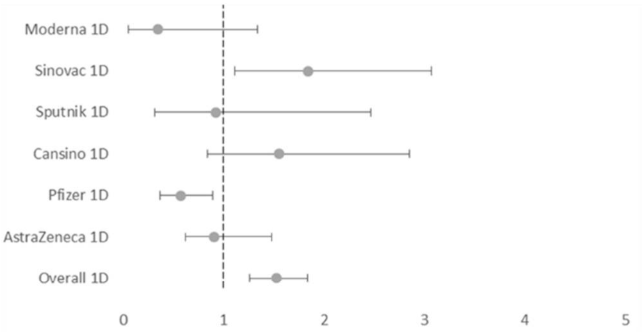

	OR	95% CI	p
Moderna 1D	0.34	0.052 to 1.339	0.1759
Sinovac 1D	1.84	1.107 to 3.065	0.0185
Sputnik 1D	0.92	0.312 to 2.466	0.8732
Cansino 1D	1.54	0.837 to 2.849	0.1598
Pfizer 1D	0.57	0.369 to 0.891	0.0136
AstraZeneca 1D	0.92	0.616 to 1.480	0.8383
Overall 1D	1.52	1.26 to 1.83	0.0005

b)

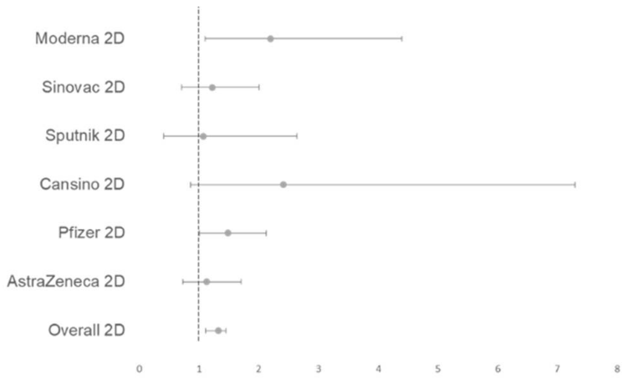

	OR	95% CI	*p*
Moderna 2D	2.200	1.104 to 4.476	**0.0260**
Sinovac 2D	1.224	0.7124 to 2.080	0.4581
Sputnik 2D	1.072	0.4130 to 2.640	0.8819
Cansino 2D	2.411	0.8557 to 7.295	0.1001
Pfizer 2D	1.484	1.031 to 2.136	**0.0333**
AstraZeneca 2D	1.131	0.7374 to 1.738	0.5731
Overall 2D	1.287	1.025 to 1.615	**0.0293**

c)

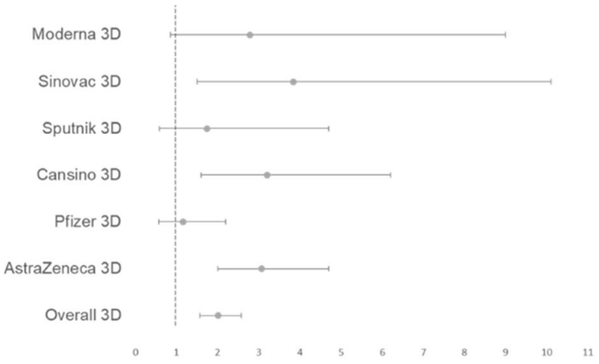

	OR	95% CI	*p*
Moderna 3D	2.791	0.8589 to 9.069	0.0801
Sinovac 3D	3.837	1.520 to 10.11	**0.0047**
Sputnik 3D	1.739	0.5811 to 4.788	0.2937
Cansino 3D	3.204	1.663 to 6.213	**0.0005**
Pfizer 3D	1.156	0.5742 to 2.221	0.6718
AstraZeneca 3D	3.072	2.019 to 4.724	**<0.0001**
Overall 3D	2.016	1.579 to 2.576	<0.0001

d)

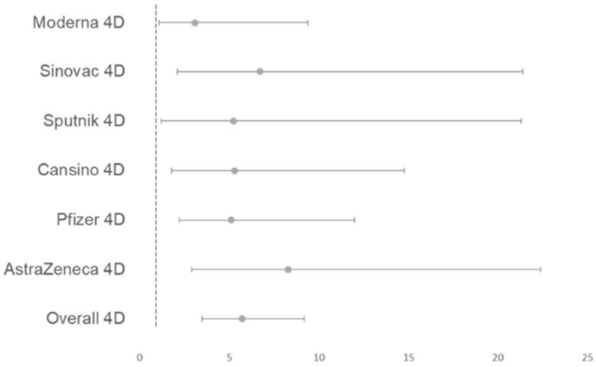

	OR	95% CI	*p*
Moderna 4D	3.196	1.089 to 9.424	**0.0330**
Sinovac 4D	6.777	2.162 to 21.40	**0.0003**
Sputnik 4D	5.236	1.290 to 21.38	**0.0168**
Cansino 4D	5.383	1.851 to 14.83	**0.0008**
Pfizer 4D	5.183	2.211 to 12.01	**<0.0001**
AstraZeneca 4D	8.373	2.979 to 22.43	**<0.0001**
Overall 4D	5.623	3.498 to 9.038	**<0.0001**

Supplementary Figure S2. The enhanced probability for menstrual cycle anomalies with dose accumulation is independent from the type of vaccine received. The odds ratios and confidence intervals for increased incidence of menstrual cycle alterations in relation to individual vaccine brands were measured after the first (a), second (b), third (c) and fourth (d) doses.

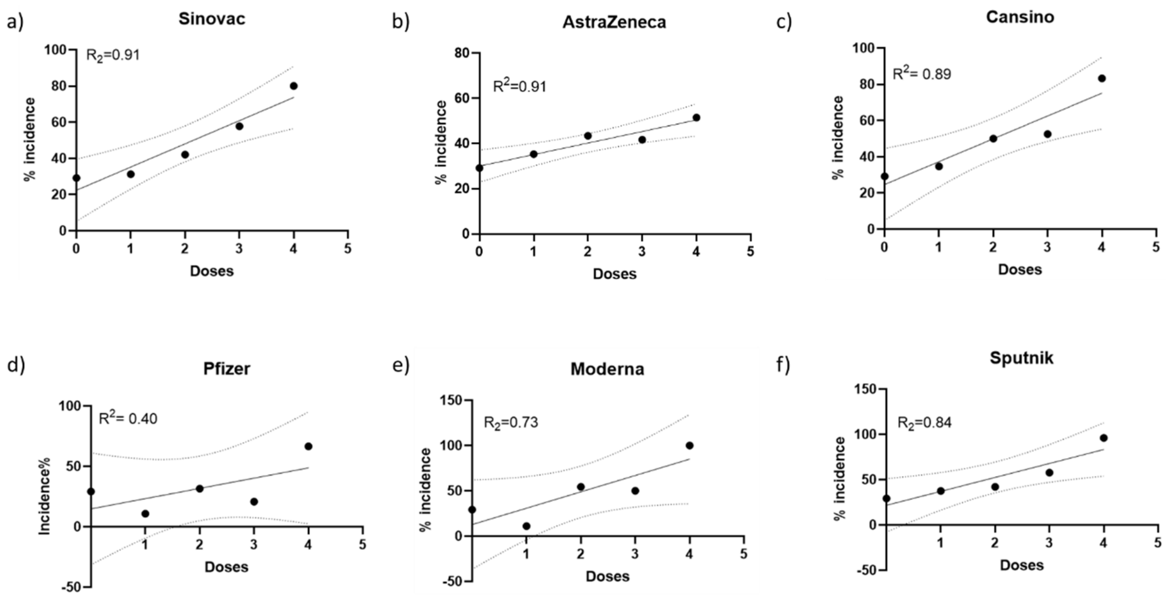

Supplementary Figure S3. Individual tendency for vaccine-brands in the increased incidence of menstrual cycle alterations with dose accumulation. A linear regression with a 95% confidence interval to describe the tendency to induce enhanced incidence of the MCA with dose accumulation was calculated for each vaccine brand, including Sinovac (a), AstraZeneca (b), Cansino (c), Pfizer (d), Moderna (e), and Sputnik (f).

Supplementary Table S2. Sensitivity analysis excluding polycystic ovary syndrome.

Dose number	*Odds ratio*	*Confidence interval at 95%*	*p value*
1st	1.781	0.9170 to 3.434	0.1100
2nd	1.426	0.7141 to 2.846	0.3176
3rd	2.698	1.343 to 5.151	**0.0040**
4th	3.273	1.219 to 8.153	**0.0205**

Supplementary Table S3. Sensitivity analysis excluding subjects using hormonal birth control.

Dose number	*Odds ratio*	*Confidence interval at 95%*	*p value*
1st	1.221	0.5862 to 2.468	0.7210
2nd	1.254	0.6256 to 2.569	0.5927
3rd	2.188	1.061 to 4.392	**0.0317**
4th	3.583	1.105 to 10.04	**0.0346**

Hospital Español de Pachuca Research Group

Writing committee
María José Rueda-Medécigo*, Adalberto León del Ángel*, Lupita Escudero-Roque*, Alejandro Maldonado del Arenal*, and Ana Elena Escorcia-Saucedo*

Operations committee
Roque D. Licona Meníndez, Elías Emanuel Licona Venegas, Karla Y. Hernández-Skewes, Daniela Pérez-Ortega, Fernanda Romero-Lechuga, Geraldine López Zaldívar, José Alberto Martín Ramírez Sierra, Mariana Paola Carmona Olivarez, Rozana Reyes-Gámez, Carolina Chavarría-Noya, Iván Karel Sánchez-Hernández, Karla García Callejas, Estephany Rodríguez Segura, Sergio Hernández Islas, Daniela Montaño Olmos, Honorio Pérez-Baca, Lizeth Castro Vite, Gabriel Romero-Lopez, Jonathan Samuel Rodríguez-Peña, Vanessa Montzerrat Magos Ramírez, Jeffrey Hernández, Andrea Reyes Torres, Diana Laura Escamilla Lorenzo, Sergio Paul Cruz Arteaga, Brenda Roxana Montiel Martínez, Alan Francisco López Gómez, Aldo Otilio Islas-Sánchez, Joselin Merida, Ernesto Nieto-Torres, Christiaan Jardinez, Edith Jiménez-Muñoz, and Pablo Antonio López Pérez.

Data curation committee
Shania Marlenne Quintanar-Trejo and Manuel Ortega Sánchez.

Statistics committee
María José Rueda-Medécigo, Alberto N. Peón

Design and supervision committee
Roque D. Licona-Meníndez¶, Alberto N. Peón¶

*Joint first authors, ¶Joint corresponding authors
